# Disease progression in women with X-linked adrenoleukodystrophy is slow

**DOI:** 10.1186/s13023-019-1008-6

**Published:** 2019-02-07

**Authors:** Irene C. Huffnagel, Marcel G. W. Dijkgraaf, Georges E. Janssens, Michel van Weeghel, Björn M. van Geel, Bwee Tien Poll-The, Stephan Kemp, Marc Engelen

**Affiliations:** 1 Department of Pediatric Neurology/Emma Children’s Hospital, Academic Medical Center, University of Amsterdam, Amsterdam, The Netherlands; 2 Department of Clinical Epidemiology, Biostatistics and Bio-informatics, Academic Medical Center, University of Amsterdam, Amsterdam, The Netherlands; 3 Laboratory Genetic Metabolic Diseases, Academic Medical Center, University of Amsterdam, Amsterdam, The Netherlands; 4 Department of Neurology, NoordWest Ziekenhuisgroep, Alkmaar, The Netherlands

**Keywords:** Adrenoleukodystrophy, Women, Spinal cord disease, Progression, Biomarkers

## Abstract

**Background:**

Over 80% of women with X-linked adrenoleukodystrophy (ALD) develop spinal cord disease in adulthood for which treatment is supportive only. For future clinical trials quantitative data on disease progression rates are essential. Moreover, diagnosis can be challenging in ALD women, as the most important diagnostic biomarker is normal in 15–20%. Better biomarkers are needed. The purpose of this single centre cross-sectional follow-up study in women with ALD was to assess whether Expanded Disability Status Scale (EDSS), AMC Linear Disability Scale (ALDS) and Short Form (36) Health Survey (SF-36) can detect disease progression and to model the effect of age and duration of symptoms on the rate of progression. Moreover, we performed a pilot study to assess if a semi-targeted lipidomics approach can identify possible new diagnostic biomarkers.

**Results:**

In this study 46 women (baseline clinical data published by our group previously) were invited for a follow-up visit. Newly identified women at our center were also recruited. We analysed 65 baseline and 34 follow-up assessments. Median time between baseline and follow-up was 7.8 years (range 6.4–8.7). Mean age at baseline was 49.2 ± 14.2 years, at follow-up 55.4 ± 10.1. EDSS increased significantly (+ 0.08 points/year), but the other outcome measures did not. Increasing age and duration of symptoms were associated with more disability. For the pilot study we analysed plasma of 20 ALD women and 10 controls with ultra-high performance liquid chromatography coupled to high-resolution mass spectrometry, which identified 100 potential biomarker ratios with strong differentiating properties and non-overlapping data distributions between ALD women and controls.

**Conclusions:**

Progression of spinal cord disease can be detected with EDSS, but not with ALDS or SF-36 after a follow-up period of almost 8 years. Moreover, age and the duration of symptoms seem positively associated with the rate of progression. Although a significant progression was measurable, it was below the rate generally conceived as clinically relevant. Therefore, EDSS, ALDS and SF-36 are not suitable as primary outcome measures in clinical trials for spinal cord disease in ALD women. In addition, a semi-targeted lipidomics approach can identify possible new diagnostic biomarkers for women with ALD.

**Electronic supplementary material:**

The online version of this article (10.1186/s13023-019-1008-6) contains supplementary material, which is available to authorized users.

## Background

The rare inborn error of metabolism X-linked adrenoleukodystrophy (ALD, Online Mendelian Inheritance in Man entry number 300100) affects both men and women [1]. In men, the clinical spectrum includes progressive spinal cord disease in all (‘adrenomyeloneuropathy (AMN)’), primary adrenal insufficiency in 80% and cerebral inflammatory disease (‘cerebral ALD’) in 60% [2–4]. As ALD is an X-linked disease, women were previously considered asymptomatic carriers. It is now known that even though adrenal insufficiency and cerebral disease occur in less than 1% of women, more than 80% eventually develop progressive spinal cord disease [5, 6]. Although both men and women develop spinal cord disease, there are differences. In women the onset of spinal cord disease is usually later in life. Furthermore, although there are no prospective studies that have evaluated this systematically, progression is considered to be slower [5]. Current treatment options for spinal cord disease are merely supportive, however, new curative therapies are under development [1]. As ALD is a rare disease, the number of patients who can participate in clinical trials is limited. It would increase the number of patients substantially, and thus speed up drug development, if both men and women could participate, despite their clinical differences.

If women with ALD are to participate in clinical trials, progression rate of spinal cord disease and factors that determine this rate of progression need to be identified. Outcome measures usable in clinical trials should be sensitive enough to measure clinical deterioration in women over a reasonable period of time, yet they should also be clinically relevant. Previous cross-sectional research has shown that women with ALD who have spinal cord disease can be clearly distinguished from those who do not, using various clinimetric scales. These scales include the Japanese Orthopaedic Association (JOA), the Severity Score system for Progressive Myelopathy (SSPROM), the Expanded Disability Status Scale (EDSS) and the AMC Linear Disability Scale (ALDS) [5, 6]. The quality of life questionnaire Short Form (36) Health Survey (SF-36) has shown a similar trend [5]. Habekost et al. [7] provided the first longitudinal data for the JOA and SSPROM, however, change on these scales was minimal over a mean observation period of 9 ± 3 months and therefore probably not practical for use in clinical trials. Recently, Schirinzi et al. [8] illustrated clinical change (+ 0.24/year) on the Adult ALD Clinical Score in 19 symptomatic women with ALD over an observation period of 3.5 ± 2.1 years. Follow-up data from large cohorts on other outcome measures is currently unavailable. Similarly, no information exists on factors that influence the rate of progression of spinal cord disease in women with ALD, but we hypothesize that age and duration of symptoms might influence progression rate, as a positive correlation has been identified between (1) symptomatic status and age, and (2) duration of symptoms and gait disorder severity [5, 6, 8, 9].

In addition to clinical differences in spinal cord disease between men and women with ALD there is a biochemical difference. Both have a mutation in the *ABCD1* gene, which encodes the ALD protein (ALDP), a peroxisomal membrane protein essential in the beta-oxidation of straight-chain very long-chain fatty acids (VLCFA; ≥22 carbon atoms) [10, 11]. Measurement of total VLCFA in plasma (specifically C26:0, the C26:0/C22:0 ratio and the C24:0/C22:0 ratio) is diagnostic in men with a nearly 100% sensitivity [12–14]. In 15–20% of women however, VLCFA levels are in the normal range [5, 14]. In the absence of elevated VLCFA an ALD diagnosis can be made by identifying a known pathogenic *ABCD1* mutation or by time consuming functional studies [15]. Recently our group reported that 1-hexacosanoyl-2-lyso-sn-3-glycero-phosphorylcholine (C26:0-lysoPC) is a better diagnostic biomarker in women than C26:0 [16]. C26:0-lysoPC levels were elevated in all 49 women even though C26:0 was not. Unfortunately, the difference between the maximum control C26:0-lysoPC level and the minimum patient C26:0-lysoPC level was small, warranting the need for a superior discriminating biomarker. If such a biomarker was identified, this would enable timely diagnosis for women with normal VLCFA levels and *ABCD1* variants of unknown significance. Semi-targeted lipidomics, an ultra-high performance liquid chromatography coupled to high-resolution mass spectrometry (UPLC-HRMS), allows the detection of over 10,000 lipid derivatives in one screen [17, 18]. If these lipids can distinguish between relevant clinical groups, for instance patients and controls, they could prove to be better diagnostic biomarkers.

The purpose of this follow-up study in women with ALD was to assess whether EDSS, ALDS and SF-36 can detect progression of spinal cord disease and to model the effect of age and the duration of symptoms of spinal cord disease on the rate of progression. Moreover, we performed a pilot study to assess if a semi-targeted lipidomics approach can identify possible new diagnostic biomarkers for ALD in women.

## Materials and methods

### Follow-up study

#### Study design and subjects

This was a single centre cross-sectional follow-up study. Baseline clinical data of 46 women with ALD have been reported by our group previously [5]. All women were invited for a follow-up visit. The visit included one hospital visit with fasted venous blood sampling, neurological history and examination and questionnaires. To expand the cohort newly identified women at our center (Academic Medical Center, Amsterdam, The Netherlands), who had an ALD diagnosis based on elevated VLCFA levels in plasma and/or a *ABCD1* mutation, were also recruited from 2015 to 17. Clinical data of these women were pooled with the previously reported baseline assessments (*n* = 46). A notification of the study was recorded on the Dutch ALD patient organization website to reach patients who did not visit our centre. Women unable to visit the hospital or suffering from neurological co-morbidity were excluded from participation. The local Institutional Review Board approved the study protocol (METC2015_079). Written informed consent was obtained from all participants.

#### Clinical assessment

Women were considered symptomatic if they had symptoms and signs of spinal cord disease [5]. Briefly, symptoms were assessed by evaluating the presence of a gait disorder, urge incontinence for urine or feces and sensory complaints. Women were considered to have a gait disorder if their maximum walking distance was limited. Sensory complaints were considered present if there was numbness or paresthesia in the lower extremities. Neurological examination included assessment of muscle strength, spasticity, reflexes and sensation. Brisk reflexes (at least three beats of clonus) or pathological plantar reflexes were considered abnormal. Sensation was assessed twice. First, sensory examination was performed as previously described [5]. Sensation was considered abnormal if there was a reduced sensation of touch, pin prick, proprioception or vibration. Vibration threshold was measured binary (present, not present) with a tuning fork (64 Hz) at the hallux. Second, an enhanced sensory examination was scored. Temperature was added to the assessment and vibration threshold was measured semiquantitatively with a Rydel-Seiffer tuning fork at the hallux and internal malleolus [19].

EDSS scores were scored separately by physicians IH and ME based on the documented history and examination [20, 21]. The EDSS ranges from 0.0 (normal) to 10.0 (death). Scores were compared and if different, these measurements were discussed until a consensus was reached. The ALDS is a survey focused on disability during activities of daily life. The units are regression coefficients (logits) and were linearly transformed for interpretation, ranging from 10 (most disability) to 89.47 (least disability) [22, 23]. SF-36 values were compared with norm values for the Dutch population and corrected for gender and age. Eight subdomains were calculated; physical functioning, role limitations due to physical problems, bodily pain, general health perceptions, vitality, social functioning, role limitations due to emotional problems and mental health. Values were expressed as Z-scores and ranged from − 4 (lowest quality of life) to + 4 (highest quality of life). Two summary scores were also composed; the physical component summary and mental component summary. These scores were linearly transformed and ranged from 0 (lowest quality of life) to 100 (highest quality of life) with a mean of 50 and a standard deviation of 10 [24, 25].

### Clinimetric evaluation

There is no “gold standard” for measuring disability. To get a sense of the clinimetric characteristics of the outcome measures used we evaluated clinical validity, construct validity and the presence of a ceiling and floor effect at baseline [23]. Clinical validity was measured by evaluating whether the outcome measures could distinguish between clinical groups. Firstly, it was assessed if the outcome measures could distinguish between symptomatic and asymptomatic women, and secondly if they could distinguish between women with unrestricted walking, restricted walking and walking with an aid. Construct validity was determined by estimating the correlation between measures assessing the same health concept. We hypothesized that measures that assess physical functioning would correlate well, in contrast to physical and mental scales [5]. Ceiling and floor effects were assessed by reporting the number of patients who reached the maximum or minimum score.

### Statistical analysis

The clinical data was analysed with IBM SPSS statistics (version 24). Outcome measures were reported as means with standard deviations (normally distributed continuous data) and as medians with ranges (non-normally distributed continuous data). Depending on the distribution, differences between two groups were assessed with independent sample Student’s t-tests or Mann Whitney U tests. Differences between more than two groups were assessed with ANOVA (normally distributed data) and Kruskal Wallis tests (non-normally distributed data). Correlations were calculated with Pearson’s correlation (normally distributed data) or Spearman’s correlation (non-normally distributed data). A *p* value < 0.05 was considered significant. If a Bonferroni correction was applied for multiple comparisons the significance level was reported separately.

Global progression rates per year were estimated using outcome measures not adjusted for covariates. Disease progression between baseline and follow-up adjusted for covariates was analysed with generalized linear mixed models. This approach allows for inclusion of women with only one examination. Separate models were made for EDSS, ALDS and the subdomains of the SF-36 which could significantly differentiate between symptomatic and asymptomatic women at baseline. A covariance structure with the lowest Bayesian information criterion value was assumed. In all models timing of assessment was included as a fixed effect and subject as a random effect. Depending on the outcome measure, age at examination and/or the duration of symptoms was included as a fixed effect. The duration of symptoms of spinal cord disease was categorized as either asymptomatic, symptoms up to 10 years, or symptoms for more than 10 years. Duration of symptoms was categorized because longer disease duration is associated with less accurate recall of onset date [26]. Models were run three times. First, including all baseline and follow-up assessments (main analysis). Second, to assess what the effect was of adding women with only one assessment, including only women with both a baseline and a follow-up assessment (subgroup analysis 1). Third, including only women who were symptomatic at baseline, or who became symptomatic during follow-up (subgroup analysis 2).

### Lipidomics study

#### Sample collection

For the pilot study we included fasted plasma data of 20 women with ALD. First, we selected five women with ALD with either a plasma C26:0 level or a C26:0/C22:0 ratio within the normal plasma C26:0 level (1.40 ± 0.40 (range 0.72–2.20)) or C26:0/C22:0 ratio (0.023 ± 0.005 (range 0.015–0.033)). Second, we selected 15 women with ALD with an elevated plasma C26:0 level and C26:0/C22:0 ratio. Ten fasted plasma samples from healthy adult females were used as controls.

#### Lipidomics

Lipids were extracted using a single-phase extraction. A defined amount of internal standards dissolved in 120 μL of chloroform/methanol (1:1, *v*/v), and 1.5 mL of chloroform/methanol (1:1, v/v) was added to 20 μL plasma. The internal standards mixture consisted of: 0.5 nmol diglycerides (DG(14:0/14:0)), 0.5 nmol triglycerides (TG(14:0/14:0/14:0)), 0.5 nmol cholesterol ester (CE(14:0)), 0.1 nmol cardiolipin (CL(14:0/14:0/14:0/14:0)), 0.2 nmol bis(monoacylglycero)phophate (BMP(14:0/14:0)), 2.0 nmol phosphatidylcholine (PC(14:0/14:0)), 0.1 nmol phosphatidylglycerol (PG(14:0/14:0)), 5.0 nmol phosphatidylserine (PS(14:0/14:0)), 0.5 nmol phosphatidylethanolamine (PE(14:0/14:0)), 0.5 nmol phosphatidic acid (PA(14:0/14:0)), 0.5 nmol phosphatidylinositol (PI(8:0/8:0)), 2.0 nmol sphingomyelin (SM(d18:1/12:0)), 0.02 nmol lysophosphatidylglycerol (LPG(14:0)), 0.1 nmol lysophosphatidylethanolamine (LPE(14:0)), 0.5 nmol lysophosphatidylchloline (LPC(14:0)) and 0.1 nmol lysophosphatidic acid (LPA(14:0)) (Avanti Polar Lipids). The mixture was sonicated in a water bath (5 min) and centrifuged (4 °C, (16,000×g, 5 min). The liquid phase was transferred to a glass vial and evaporated under a stream of nitrogen at 60 °C. Subsequently, the residue was dissolved in 150 μL of chloroform/methanol (9:1, *v*/v), and analysed using an ultra-high performance liquid chromatography coupled to high-resolution mass spectrometry (UPLC-HRMS) system.

The UPLC-HRMS system consisted of an Ultimate 3000 binary HPLC pump, a vacuum degasser, a column temperature controller, and an auto sampler (Thermo Scientific). For normal phase, 2.5 μL lipid extract was injected onto a LiChroCART 250–4 LiChrospher® Si 60 (5 μm) (Merck) maintained at 25 °C. Lipids were separated from interfering compounds by a linear gradient consisting of solution A (methanol/water, 85:15, *v*/v) and solution B (chloroform/methanol, 97:3, v/v). Solutions A and B contained 5 and 0.2 ml of 25% (*v*/v) aqueous ammonia per liter of eluent, respectively. The gradient (0.3 ml/min) was as follows: T = 0–1 min: 10%A; T = 1–4 min: 10%A-20%A; T = 4–12 min: 20%A-85%A; T = 12–12.1 min: 85%A - 100%A; T = 12.1–14.0 min: 100%A; T = 14–14.1 min: 100%A-10%A and T = 14.1–15 min: 10%A. For reverse phase, 5 μL lipid extract was injected onto a ACQUITY UPLC HSS T3, 1.8 μm particle diameter (Waters) maintained at 60 °C. Lipids were separated from interfering compounds by a linear gradient consisting of solution A (methanol/water, 40:60, *v*/v) and solution B (methanol/isopropanol, 10:90, v/v). Solutions A and B both contained 0.1% formic acid and 10 mM ammonia. The gradient (0.4 ml/min) was as follows: T = 0–1 min: 100%A; T = 1–16 min: 80%A; T = 16–20 min: 0%A; T = 20–20.1 min: 0%A; T = 20.1–21.0 min: 100%A. A Thermo Scientific Q Exactive Plus Orbitrap mass spectrometer was used in the negative and positive electrospray ionization mode. Nitrogen was used as the nebulizing gas, spray voltage 2500 V, capillary temperature 256 °C, S-lens radio frequency level 50, auxiliary gas flow rate 11 a.u., auxiliary gas heater temperature 300 °C, sheath gas flow rate 48 a.u., sweep gas flow rate 2 a.u.. Mass spectra of lipid molecular species were obtained, in both the negative and positive mode, by continuous scanning from m/z 150 to 2000 with a resolving power of 280,000 full width at half maximum (FWHM).

#### Bioinformatics and biomarker discovery

The statistical programing language R (http://www.r-project.org) was used to analyse the lipidomics data. Pre-processing was performed with an in-house metabolomics pipeline [18]. To generate a list of candidate biomarkers we firstly defined lipid levels as the relative abundance of each lipid normalized to the corresponding internal standard used for that lipid class. Normalized lipid levels were visualized in a Volcano plot [27]. The vertical axis contains the *p*-value (−log10) from t-tests between ALD women and controls, and the horizontal axis the fold change (log2) between ALD women and controls. Lipids with a *p* value < 0.001 and an absolute fold change (log2) larger than one were considered potentially interesting biomarkers. Secondly, lipids were ranked for differential abundance based on their variable importance of projection (VIP) scores. The VIP scores were constructed using partial least squares regression discriminant analysis (PLS-DA) using the R package ‘mixOmics’ [28]. Thirdly, the top 250 lipids ranked for differential abundance were selected and pairwise Pearson correlations were calculated between all lipids using the R package ‘corrplot’ [29]. Lastly, a list of the top 100 biomarker ratios were selected based on the most anti-correlating hits that had non-overlapping data distributions between women with ALD and controls.

## Results

### Follow-up study

#### Clinical characteristics of the cohort

**Fig. 1:**
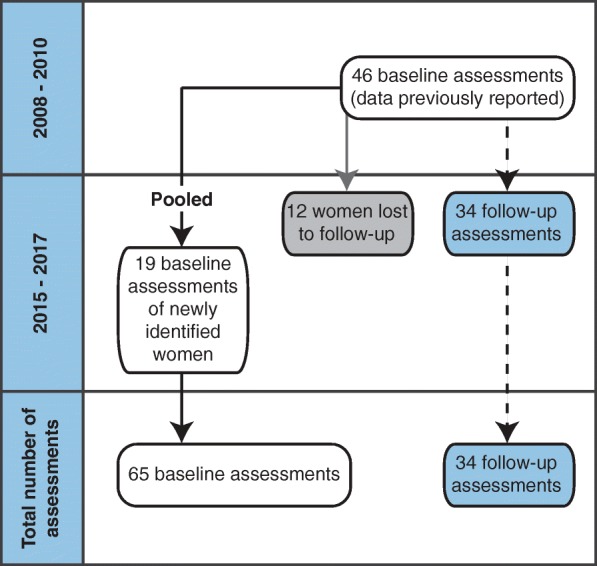
Flowchart of subject inclusion

The 46 women with ALD previously included were contacted for the follow-up visit [5]. Thirty-four (74%) agreed to an additional visit. Twelve (26%) were lost to follow-up. Reasons for withdrawal included death not related to ALD (1/12), unknown contact information (1/12), inability to visit the hospital (2/12), malignancy requiring treatment during the assessment period (1/12) and unwillingness to participate (7/12). A logistic regression model to evaluate the effects of age and EDSS score at baseline assessment on the likelihood that women were lost to follow-up, suggested random loss to follow-up (Table 1). Nineteen newly identified women with ALD also agreed to participate. No women were excluded due to neurological co-morbidity. Subject inclusion is visualized in Fig. 1.

**Table 1:** Logistic regression predicting loss to follow-up likelihood with age and EDSS score at baseline

	B	SE	Wald	dF	P value	Odds ratio	95% Confidence interval for odds ratio	
							Lower	Upper
Age at baseline	− 0.02	0.036	0.318	1	0.573	0.980	0.914	1.051
EDSS score at baseline	0.363	0.241	2.268	1	0.132	1.437	0.896	2.305
Constant	−1.141	1.454	0.616	1	0.433	0.319		

B = coefficient; dF = degrees of freedom; EDSS = Expanded Disability Status Scale; SE = standard error

**Fig. 2:**
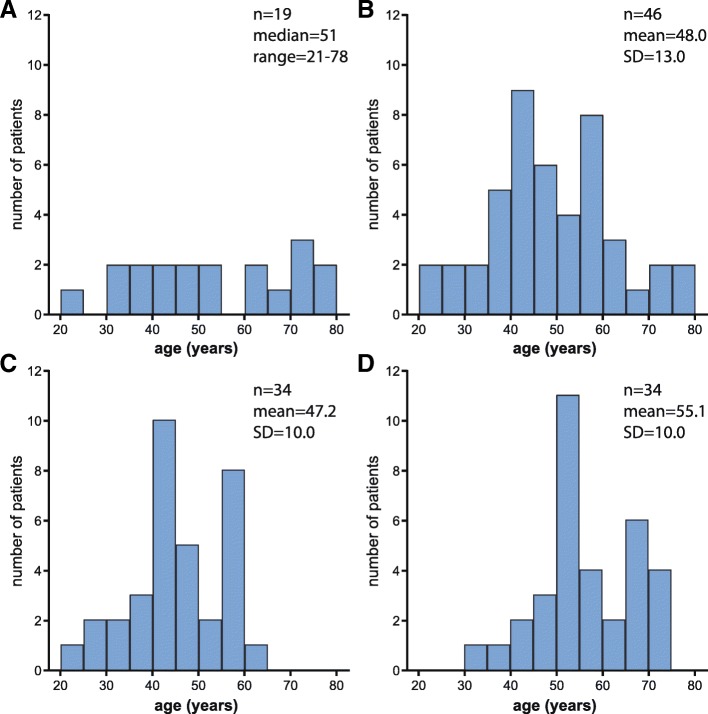
Age distribution. **a** Age (years) distribution of the 19 newly identified women. **b** Age (years) distribution of the previously published cohort of 46 women. **c** Age (years) distribution at baseline of the 34 women with two measurements.**d** Age (years) distribution at follow-up of the 34 women with two measurements

Sixty-five baseline and 34 follow-up assessments were available for analysis. Clinical characteristics of the cohort are listed in Table 2. In addition, the age distribution per time point and group of women (19 newly identified women, 46 previously reported women and 34 women with follow-up assessments respectively) is visualized in Fig. 2. The enhanced sensory examination did not label additional patients as symptomatic. During follow-up 8/34 (24%) women became symptomatic. Twelve women developed incontinence for urine during the follow-up period, six incontinence for feces and 12 sensory complaints. There were solely two women that developed spasticity during this period, but 11 developed abnormal sensation at examination, 10 weakness and an additional seven developed pathological reflexes. Of the 37 symptomatic women at baseline 22 (59%) had symptoms for over 10 years. Individual mutations, symptoms and signs are listed in Additional file 1.

**Table 2:** Summary of clinical data and outcome measures at baseline

	N	Mean ± SD / median (range) / proportion
*Clinical characteristics of the cohort*		
Follow-up time in years	34	7.8 (6.4–8.7)
Symptomatic (baseline)	65	37 (57%)
Symptomatic (follow-up)	34	27 (79%)
Conversion to symptomatic during follow-up period	34	8 (24%)
Walking (baseline) - unrestricted	62	40 (65%)
- restricted	62	14 (23%)
- with an aid	62	8 (13%)
Change in walking status during follow-up	32	3 (0.1%)
Age in years (baseline)	65	49.2 ± 14.2
- Age youngest symptomatic woman	1	36
- Age oldest asymptomatic woman	1	73
- Age youngest woman with aided walking	1	38
- Age oldest woman with unrestricted walking	1	74
*Outcome measures at baseline*		
EDSS	63	2.5 (0–6)
ALDS	64	89.47 (71.92–89.47)
SF-36: Physical functioning	64	0.22 (−3.69–1.16)
SF-36: Role limitations due to physical problems	64	0.52 (−2.55–1.63)
SF-36: Bodily pain	64	0.12 (− 3.11–1.50)
SF-36: General health perceptions	64	−0.15 (− 3.63–1.85)
SF-36: Vitality	63	−0.14 (− 2.48–1.58)
SF-36: Social functioning	64	0.24 (− 3.02–1.09)
SF-36: Role limitations due to emotional problems	64	0.55 (− 2.77–0.95)
SF-36: Mental health	63	0.33 (−1.79–1.54)
SF-36: Physical component summary	63	49.37 (17.26–62.36)
SF-36: Mental component summary	63	53.89 (35.89–66.30)

The EDSS ranges from 0 (normal) to 10 (death). The ALDS scores are regression coefficients which were linearly transformed for interpretation, ranging from 10 (highest level of disability) to 89.47 (lowest level of disability). SF-36 values were compared with norm values for the Dutch population and corrected for gender and age. The SF-36 subdomain scores are expressed in Z scores, ranging from −4 (lowest quality of life) to + 4 (highest quality of life). The SF-36 summary scores were linearly transformed to a range from 0 (lowest quality of life) to 100 (highest quality of life), with a mean of 50 and standard deviation of 10

ALDS = AMC Linear Disability Scale; EDSS = Expanded Disability Status Scale; SD = standard deviation; SF-36 = Short Form (36) Health Survey

Median EDSS score at baseline was 2.5 (range 0.0–6.0), indicating minimal disability in two functional systems. Clinically, this could represent rare urinary incontinence and mild sensory deficits. An EDSS score of 6.0 represents the necessity of assisted walking. Median ALDS score was 89.47 (range 71.92–89.47). Median SF-36 domain scores for general health perceptions, vitality and physical component summary were just below average, but the others were just above. Individual scores per outcome measure are listed in Additional file 2, a summary in Table 2.

### Clinimetric evaluation

The clinimetric evaluation was conducted using the baseline assessments.

### Clinical validity

There was a significant difference in score distributions between symptomatic and asymptomatic women for the EDSS, ALDS, physical functioning and physical component summary, as assessed with Mann-Whitney U tests (Table 3). Kruskal-Wallis tests were used to assess whether there were differences in scores between the three ambulation groups (unrestricted walking, restricted walking and walking with an aid) (Table 4). The distributions of EDSS, ALDS, physical functioning, role physical, bodily pain, general health perceptions and physical component summary were significantly different between groups. Post hoc pairwise comparisons revealed a significant difference between unrestricted walking and walking an aid for EDSS, ALDS, physical functioning, bodily pain and physical component summary. Likewise, post hoc pairwise comparisons revealed a significant difference between unrestricted walking and restricted walking for EDSS, ALDS, physical functioning and physical component summary. Not a single outcome measure revealed a significant difference between restricted walking and walking with an aid post hoc.

**Table 3:** Clinical validity – symptomatic and asymptomatic

	N	U	Symptomatic		Asymptomatic		P value
			*N*	*Mean Rank*	*N*	*Mean rank*	
EDSS	63	929.000	37	44.11	26	14.77	< 0.0005 *
ALDS	64	231.000	36	24.92	28	42.25	< 0.0005 *
SF-36: Physical functioning	64	294.500	36	26.68	28	39.98	0.005 *
SF-36: Role physical	64	379.500	36	29.04	28	36.95	0.09
SF-36: Bodily pain	64	360.000	36	28.50	28	37.64	0.051
SF-36: General health perceptions	64	370.000	36	28.78	28	37.29	0.070
SF-36: Vitality	63	512.000	35	32.63	28	31.21	0.761
SF-36: Social functioning	64	496.000	36	32.28	28	32.79	0.914
SF-36: Role emotional	64	588.000	36	34.83	28	29.50	0.249
SF-36: Mental health	63	517.000	35	32.77	28	31.04	0.709
SF-36: Physical component summary	63	282.000	35	26.06	28	39.43	0.004 *
SF-36: Mental component summary	63	614.000	35	35.54	28	27.57	0.086

Mann-Whitney U tests were run to assess if there were differences in scores between symptomatic and asymptomatic women at baseline. * indicates a significant p value. ALDS = AMC Linear Disability Scale; EDSS = Expanded Disability Status Scale; N = number of patients; Role emotional = role limitations due to emotional problems; Role physical = role limitations due to physical problems; SF-36 = Short Form (36) Health Survey; U = Mann-Whitney U statistic

**Table 4:** Clinical validity – unrestricted, restricted and aided walking

	N	H	Unrestricted walking		Restricted walking		Walking with aid		P value
			*N*	*Mean Rank*	*N*	*Mean rank*	*N*	*Mean rank*	
EDSS	62	33.378	40	22.44	14	42.25	8	58	< 0.0005 *
ALDS	61	28.984	40	38.27	14	21.89	7	7.64	< 0.0005 *
SF-36: Physical functioning	61	20.216	40	37.98	14	21.82	7	9.50	< 0.0005 *
SF-36: Role physical	61	8.220	40	35.65	14	23.21	7	20.00	0.016 *
SF-36: Bodily pain	61	7.245	40	35.08	14	26.11	7	17.50	0.027 *
SF-36: General health perceptions	61	6.626	40	35.00	14	25.71	7	18.71	0.036 *
SF-36: Vitality	60	2.967	39	33.23	14	24.11	7	28.07	0.227
SF-36: Social functioning	61	1.960	40	32.79	14	30.00	7	22.79	0.375
SF-36: Role emotional	61	0.764	40	29.60	14	33.25	7	34.50	0.683
SF-36: Mental health	60	0.012	39	30.60	14	30.07	7	30.79	0.994
SF-36: Physical component summary	60	15.527	39	36.72	14	22.14	7	12.57	< 0.0005 *
SF-36: Mental component summary	60	2.278	39	28.90	14	30.36	7	39.71	0.320

Kruskal-Wallis tests were run to assess if there were differences in scores between the three ambulation groups at baseline. * indicates a significant *p* value. ALDS = AMC Linear Disability Scale; EDSS = Expanded Disability Status Scale; H = Kruskal-Wallis H statistic; N = number of patients; Role emotional = role limitations due to emotional problems; Role physical = role limitations due to physical problems; SF-36 = Short Form (36) Health Survey

### Construct validity

A Bonferroni correction was applied for multiple comparisons. Correlations were considered significant if below 0.0042 (2-tailed). The scores that measured physical functioning (EDSS, ALDS, physical functioning and physical component summary) correlated strongly (correlation coefficient > 0.411, *p* <  0.001) with each other but poorly with mental health and mental component summary. Likewise, bodily pain and general health perceptions also correlated poor with mental health and mental component summary, but well with most of the other SF-36 domains. Correlation with the EDSS, however, was also poor. Details of the construct validity are presented in Additional file 3.

### Ceiling and floor effect

There was no ALD related mortality, meaning that no women had the maximum EDSS score of 10. Ten women (10/63; 15%) had the minimum EDSS score of zero. Even though the individual differences between these 10 women could not be measured with the EDSS, they scored two different scores on the ALDS, six on the physical functioning, and 10 on physical component summary.

No patients scored the maximum ALDS score and 38 women (38/64; 59%) had the minimum score of 89.47. These 38 women scored five different scores on the EDSS, 22 on the physical functioning, and 38 individual scores on the physical component summary.

No patients scored the maximum or minimum score at baseline on any of the SF-36 subdomains. Physical component summary and mental component summary were the only outcome measures for which each woman had an individual score.

### Progression rates

Table 5 lists absolute numbers of women who showed clinical disease progression (i.e. a worse score), remained stable or improved (i.e. a better score) during follow-up.

**Table 5:** Progression rates

	N	Clinical progression	Stable score	Improvement score	Baseline (range)	Follow-up (range)	Progression rates per year		
							All women	Symptomatic at baseline (*N* = 19)	Asymptomatic at baseline (*N* = 15)
EDSS	32	21	11	0	2.75 (0–6)	3.5 (1.5–6)	0.08	0.06	0.17
ALDS	34	10	17	7	89.47 (71.92–89.47)	89.47 (71.92–89.47)	0.00	0.00	0.00
SF-36: PF	34	19	1	14	0.22 (−2.66–1.16)	− 0.07 (−2.86–1.26)	0.00	−0.03	0.02
SF-36: PCS	34	21	0	13	50.11 (17.26–62.36)	49.16 (16.67–64.72)	− 0.21	− 0.17	− 0.37

Progression rates were calculated with values unadjusted for covariates. The EDSS ranges from 0 (normal) to 10 (death). The ALDS scores are regression coefficients which were linearly transformed for interpretation, ranging from 10 (highest level of disability) to 89.47 (lowest level of disability). The SF-36 subdomain physical functioning is expressed in Z scores, ranging from −4 (lowest quality of life) to + 4 (highest quality of life). The SF-36 physical component summary score was linearly transformed to a range from 0 (lowest quality of life) to 100 (highest quality of life), with a mean of 50 and standard deviation of 10. ALDS = AMC Linear Disability Scale; EDSS = Expanded Disability Status Scale; N = number of women; PCS = physical component summary; PF = physical functioning

Of the women who showed clinical progression on any of the outcome measures, five women showed progression on all four outcome measures, seven women on three outcome measures, eight women on two outcome measures and six women on one outcome measure.

Of the 11 women with a stable EDSS score during follow-up, five remained stable on the ALDS, three showed improvement and three showed clinical progression.

Of the women who improved during follow-up, three women showed improvement on three outcome measures, 10 women on two outcome measures and eight women on one outcome measure. No women showed improvement on all outcome measures.

Median annual progression rates per outcome measure are listed in Table 5. Significant clinical progression as assessed with the mixed models was measured using the EDSS outcome measure, which is discussed below. Median progression per year was 0.08 points and – as the EDSS ranges from 0.0 to 10.0 - indicates very slow progression. If only the asymptomatic women at baseline were included, this increased to 0.17 points per year. For the ALDS and SF-36 subdomain physical functioning, overall median progression rate per year was zero. For the SF-36 subdomain physical component this was − 0.21, indicating a worse score over time. The decrease in score over time was larger for the women who were asymptomatic at baseline (− 0.37) than for the symptomatic women at baseline (− 0.17).

### Modelling of progression

Disease progression between baseline and follow-up adjusted for covariates was analysed with generalized linear mixed models. When including all assessments, timing of assessment (i.e. baseline or follow-up assessment, main analysis) was a significant main effect for the EDSS outcome measure (B = − 0.169, SE = 0.049, *p* = 0.001), but not for the other outcome measures. Post hoc pairwise comparisons revealed a significant increase in EDSS score during follow-up of 0.73 points (SE = 0.25, *p* = 0.005). Moreover, asymptomatic women had a significantly lower EDSS score (− 2.75 points) than women with symptoms for more than 10 years (SE = 0.56, *p <* 0.0005). Although timing of assessment was not a significant main effect for the ALDS, indicating that there was no significant progression during the follow-up period, an increase in age was associated with a lower ALDS score and thus higher disability (B = − 0.004, SE = 0.002, *p* = 0.045). Moreover, similar to the EDSS, asymptomatic women had a significantly higher ALDS score (0.68 points) than women with symptoms for more than 10 years (SE = 0.28, *p =* 0.019). The association between duration of symptoms and disease severity was also detected for the SF-36 subdomains physical functioning and the physical component summary. Model details are listed in Table 6.

**Table 6:** Generalized Linear Mixed Model details

	Fixed effect	B	SE	P value	95% Confidence Interval	
					Lower	Upper
EDSS	Baseline^	- 0.169	0.049	0.001 *	−0.266	− 0.071
	No neurological symptoms^^	- 0.671	0.080	< 0.0005 *	−0.829	−0.512
	Symptoms 10 years or shorter^^	- 0.127	0.078	0.106	−0.281	0.028
ALDS	Baseline^	- 0.023	0.032	0.468	−0.086	0.040
	No neurological symptoms^^	0.170	0.066	0.012 *	0.038	0.302
	Symptoms 10 years or shorter^^	0.106	0.067	0.116	−0.027	0.239
	Age at examination	- 0.004	0.002	0.045 *	−0.009	− 0.000
SF-36: PF	Baseline^	- 0.003	0.028	0.912	−0.060	0.053
	No neurological symptoms^^	0.363	0.075	< 0.0005 *	0.215	0.511
	Symptoms 10 years or shorter^^	0.258	0.090	0.005 *	0.080	0.435
SF-36: PCS	Baseline^	0.010	0.033	0.765	−0.055	0.075
	No neurological symptoms^^	0.281	0.072	< 0.0005 *	0.137	0.424
	Symptoms 10 years or shorter^^	0.177	0.089	0.050 *	0.000	0.353

A higher EDSS score indicates higher disability. A lower ALDS score indicates higher disability. A lower physical functioning and physical component summary score indicates lower quality of life. ^ = Reference group is the follow-up visit; ^^ = Reference group is neurological symptoms > 10 years; * indicates a significant p value; B = coefficient; ALDS = AMC Linear Disability Scale; EDSS = Expanded Disability Status Scale; PCS = physical component summary; PF = physical functioning; SE = standard error; SF-36 = Short Form (36) Health Survey

When including only women with two assessments (*n* = 34, subgroup analysis 1, Table 7) timing of assessment remained a significant main effect for the EDSS (B = − 0.215, SE = 0.051, *p* <  0.0005). Likewise, timing of assessment was still not a significant main effect for the other outcome measures. In addition, the significant effect of age at examination and duration of symptoms on the ALDS disappeared.

**Table 7:** Generalized Linear Mixed Model subgroup analysis 1 solely including women with two assessments (*n*=34)

	Fixed effect	B	SE	P value	95% Confidence Interval	
					Lower	Upper
EDSS	Baseline^	−0.215	0.051	< 0.0005*	− 0.317	− 0.112
	No neurological symptoms^^	−0.536	0.077	< 0.0005*	−0.690	−0.382
	Symptoms 10 years or shorter^^	−0.151	0.079	0.060	−0.309	0.006
ALDS	Baseline^	−0.016	0.044	0.708	−0.104	0.071
	No neurological symptoms^^	0.159	0.097	0.107	−0.035	0.353
	Symptoms 10 years or shorter^^	0.120	0.100	0.234	−0.080	0.321
	Age at examination	−0.004	0.004	0.345	−0.013	0.005
SF-36: PF	Baseline^	−0.000	0.027	0.994	−0.053	0.053
	No neurological symptoms^^	0.286	0.107	0.009*	0.073	0.499
	Symptoms 10 years or shorter^^	0.211	0.131	0.112	−0.051	0.472
SF-36: PCS	Baseline^	0.010	0.033	0.766	−0.056	0.076
	No neurological symptoms^^	0.234	0.111	0.039*	0.013	0.454
	Symptoms 10 years or shorter^^	0.201	0.135	0.142	−0.069	0.471

A higher EDSS score indicates higher disability. A lower ALDS score indicates higher disability. A lower physical functioning and physical component summary score indicates lower quality of life. ^ = Reference group is the follow-up visit; ^^ = Reference group is neurological symptoms > 10 years; * indicates a significant p value; ALDS = AMC Linear Disability Scale; B = coefficient; EDSS = Expanded Disability Status Scale; PCS = physical component summary; PF = physical functioning. SE = standard error; SF-36 = Short Form (36) Health Survey

Moreover, when including women who were symptomatic at baseline or became symptomatic during follow-up (37 baseline assessments and 27 follow-up assessments, subgroup analysis 2, Table 8) timing of assessment was still only a significant main effect for the EDSS (B = − 0.107, SE = 0.040, *p* = 0.010). The increase in EDSS score, however, was now - although still significant - smaller (post hoc pairwise contrast 0.51 points, SE = 0.22, *p* = 0.022) compared to when all assessments were included (− 0.73 points).

**Table 8:** Generalized Linear Mixed Model subgroup analysis 2 solely including symptomatic women

	Fixed effect	B	SE	P value	95% Confidence Interval	
					Lower	Upper
EDSS	Baseline^	−0.107	0.040	0.010*	−0.188	−0.026
	No neurological symptoms^^	−0.347	0.109	0.002*	−0.564	−0.129
	Symptoms 10 years or shorter^^	−0.127	0.074	0.093	−0.276	0.022
ALDS	Baseline*	−0.020	0.045	0.663	−0.111	0.071
	No neurological symptoms^^	0.137	0.120	0.257	−0.103	0.377
	Symptoms 10 years or shorter^^	0.095	0.085	0.267	−0.075	0.266
	Age at examination	−0.009	0.004	0.021*	−0.016	−0.001
SF-36: PF	Baseline^	0.066	0.040	0.107	−0.015	0.146
	No neurological symptoms^^	0.352	0.131	0.010*	0.089	0.614
	Symptoms 10 years or shorter^^	0.263	0.105	0.015*	0.052	0.474
SF-36: PCS	Baseline^	0.048	0.041	0.245	−0.034	0.131
	No neurological symptoms^^	0.206	0.122	0.097	−0.038	0.450
	Symptoms 10 years or shorter^^	0.184	0.098	0.067	−0.013	0.380

Women who were symptomatic at baseline or who became symptomatic during follow-up were included (37 baseline assessments and 27 follow-up assessments). A higher EDSS score indicates higher disability. A lower ALDS score indicates higher disability. A lower physical functioning and physical component summary score indicates lower quality of life. ^ = Reference group is the follow-up visit; ^^ = Reference group is neurological symptoms > 10 years; * indicates a significant p value; ALDS = AMC Linear Disability Scale; B = coefficient; EDSS = Expanded Disability Status Scale; PCS = physical component summary; PF = physical functioning; SE = standard error; SF-36 = Short Form (36) Health Survey

### Lipidomics study

**Fig. 3:**
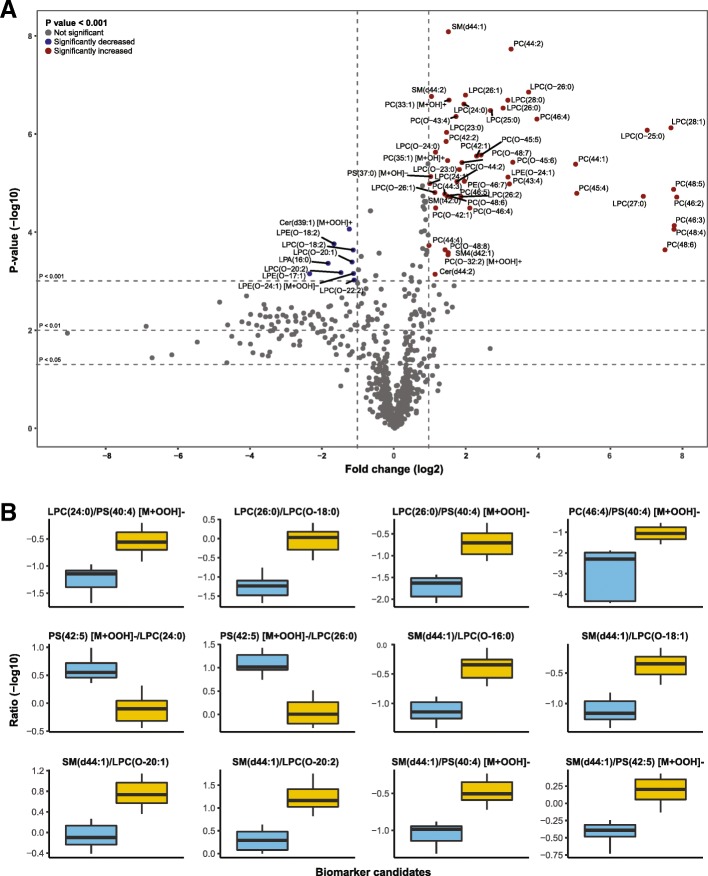
Volcano plot and biomarker ratios. **a** Volcano plot of lipid levels normalized to the corresponding internal standard. The vertical axis contains the *p*-value (−log10) from t tests between women with ALD and controls, and the horizontal axis the fold change (log2) between women with ALD and controls. Red and blue coloured dots are lipids with a *p* value < 0.001 and an absolute fold change (log2) larger than one, which were considered potentially interesting biomarkers. **b** A representative sample of the top 100 biomarker ratios with strong differentiating properties and non-overlapping data distributions between women with ALD and healthy control females. Cer = ceramide; LPA = lysophosphatidic acid; LPE = lysophosphatidylethanolamine; LPC = lysophosphatidylcholine; PC = phosphatidylcholine; PS = phosphatidyloserine; SM = sphingomyelin

A semi-targeted lipidomics analysis was performed to identify biomarkers with a better sensitivity than the conventional plasma C26:0 level and/or C26:0/C22:0 ratio. Plasma of 20 women with ALD of whom five had a plasma VLCFA level in the normal range and 15 an elevated plasma VLCFA level and 10 female controls were included. After pre-processing of the dataset, lipid levels were defined as the relative abundance of each lipid normalized to the corresponding internal standard used for that lipid class (Fig. 3a). There were 56 lipids with a *p* value < 0.001 and an absolute fold change (log2) larger than one, which were considered potentially interesting biomarkers. Of these lipids, 47 had a higher abundancy in ALD women compared to controls and nine had a lower abundancy (Fig. 3a). Overall, there was an increase in VLCFA-containing lysophospholipids, which are a glycerophospholipid subgroup containing only one fatty acid side chain, and phospholipids, which are a glycerophospholipid subgroup containing two fatty acid side chains. In more detail, the increase was detected in lysophosphatidylcholines (LPC(23:0) to LPC(28:1)), ether lysophospholipids (LPC(O-23:0) to LPC(O-26:1)), phosphatidylcholines (PC(42:1) to PC(48:6)), ether phosphatidylcholines (PC(42:1) to PC(48:8)) and sphingomyelins (SM(d44:1) and SM(d44:2). The majority of the lipid species with a lower abundancy contained long-chain fatty acids and belonged to the lysophosphatidic acid (LPA(16:0)), ether lysophosphatidylcholine (LPC(O-18:2) to LPC(O-22:2)) and ether lysophosphatidylethanolamine (LPE(O-17:1) and LPE(O-18:2)) classes.

As biomarker ratios are known to be more robust in comparison to absolute metabolite values we focussed on biomarker ratios for this pilot study. Based on the total dataset, we generated a list of ratios with the strongest anti-correlation and non-overlapping data distributions between women with ALD and controls. A representative sample of these ratios is shown in Fig. 3b. The classes of lipids included in these ratios were similar to the 56 lipids mentioned above. Although these biomarker ratios should be validated in an external cohort, they represent a candidate list of potentially good diagnostic biomarkers.

## Discussion

In the largest follow-up study in women with ALD to date, we provide evidence that during a follow-up period of almost 8 years the EDSS, but not the ALDS and SF-36, can detect progression of spinal cord disease, although this progression is below the rate that is generally considered as clinically relevant for clinical trial design [30, 31]. Moreover, age and the duration of symptoms seem positively associated with the rate of progression.

The significant progression that the EDSS detected is supported by our clinical observation that eight women became symptomatic during the follow-up period and the ambulation status (i.e. unrestricted walking, restricted walking and assisted walking) altered in three. Moreover, there were women who developed incontinence for urine, incontinence for feces and sensory complaints/abnormal sensation examination during the follow-up period. Only the EDSS detected this clinical change as a significant – albeit minor - increase in EDSS score over time with 0.73 points. In contrast to the SF-36 and ALDS, the EDSS score captures the degree of incontinence and sensory abnormalities in the final score. Interestingly, subgroup analysis 2 (with symptomatic women) also detected this progression, but the increase was smaller (+ 0.51 points). This is supported by the observed difference in annual EDSS progression rates, which were calculated with outcome measures not adjusted for covariates. For women with two assessments (*n* = 32) progression rate per year was 0.08, for women symptomatic at baseline (*n* = 19) 0.06 and women asymptomatic at baseline (*n* = 15) 0.17 (Table 5). As abnormal neurological signs in the absence of symptoms contribute to the EDSS score, this most likely reflects progression of disease in presymptomatic women. In addition, slow clinical progression on the EDSS is supported by work of Schmidt et al. (2001), who detected no progression on EDSS after 4 years in 8 women, and Habekost et al. (2015) who reported significant but slow progression on the Severity Score system for Progressive Myelopathy, a myelopathy scale that – similar to EDSS – incorporates both symptoms and abnormal neurological signs [7, 9].

Furthermore, there are theoretical considerations that reinforce the interpretation of our results. *ABCD1* deficiency with defective ALDP and subsequent VLCFA accumulation are thought to be responsible for the axonal degeneration underlying the clinically detectable spinal cord disease in ALD patients [1]. Using ALD knockout mice, Gong et al. (2017) recently showed that primed *ABCD1*-deficient microglia are likely involved in the pathophysiology of spinal cord disease in ALD [32]. Early signs of this priming are already detectable in postnatal mice [32]. Axonal degeneration probably starts early and slowly progresses during life until it becomes clinically detectable later in life. As there have been no reasons so far to assume nonlinear progression of spinal cord disease, this supports the slow clinical deterioration we observed in our cohort [7].

Nonetheless, various uncertainties in the interpretation of our results remain. The cohort was small and heterogeneous, as the youngest symptomatic woman was 36 and the oldest asymptomatic woman was 73 years of age. The use of generalized linear mixed models allowed us to increase the cohort size because it enables the inclusion of women with only one assessment. The 19 newly identified women were included as baseline assessments and not as follow-up assessments. The age distribution of these 19 women resembled the remaining cohort at baseline more than it did at follow-up (Fig. 2). Their median age, however, was slightly higher than the group with two assessments (*n* = 34). As the percentage of symptomatic women increases with age, adding the 19 women as a baseline assessment could diminish the contrast between baseline and follow-up, subsequently underestimating the progression of spinal cord disease [5]. This was not supported by subgroup analysis 1 (with women with two assessments), as timing of measurement did not become a significant main effect for other outcome measures than the EDSS - for which timing was already a significant main effect. Furthermore, there were two women who could not visit the hospital, potentially causing selection bias. If the severity of spinal cord disease was the reason for not being able to visit the hospital, that could have generated an underestimation of disease progression, as the severely affected women were then not included in the follow-up assessments. In addition, theoretically, differences in symptomatic therapy (i.e. physical therapy, spasmolytics or anticholinergic medication for urge incontinence) at baseline and follow-up could have influenced assessments. However, our clinical observation is that efficacy of these treatments is limited making it unlikely that this is a relevant confounding factor.

Although the EDSS could detect significant disease progression, the ALDS and SF-36 were not sensitive enough to detect this change. As the subgroup analyses also did not show significant change, these suggest that progression was not underestimated due to adding women with only one assessment (subgroup analysis 1) or because women that remained asymptomatic during follow-up were included (subgroup analysis 2). Clinical validity at baseline was poor for all outcome measures. Even though they could differentiate between symptomatic and asymptomatic women, they could not discriminate between symptomatic women with restricted walking and aided walking. On the other hand, while the distinction between restricted walking and aided walking is straightforward, the tipping point of when a patient converts is not. When a patients chooses to start using a walking aid is subjective and is different for each individual based on their personality traits and specific circumstances. Nevertheless, ideal outcome measures would be sensitive enough to detect differences between these groups. Although construct validity was good, inconsistencies amongst the various outcome measures remained. There were only five women who showed progression on all outcome measures and some even improved over time on the ALDS and the SF-36 subdomains physical functioning and physical component summary. As ALD is a slowly progressive neurodegenerative disease it is highly unlikely that improvement over time reflects the true natural history of spinal cord disease in women with ALD [1]. Despite somewhat poor sensitivity, analysis of the construct validity showed that measures that assess disability correlated strongly with those that assess neurological impairment. As described by us in a previous study the correlation between the physical and mental status is poor, thereby underlining the fact that quality of life can be good despite having a disability [5]. Information on other psychometric properties is lacking, and despite often being problematic in rare diseases it would be desirable to assess internal consistency and test- retest reliability in a larger cohort.

Besides significant progression of disease on EDSS, generalized linear mixed model results suggest that age and the duration of symptoms of spinal cord disease are positively associated with the rate of progression. Increasing age was associated with a higher degree of disability as assessed by the ALDS. In addition, the longer the duration of symptoms, the higher the EDSS score, the lower the ALDS score (indicating more disability) and the worse quality of life was as measured on the SF-36 subdomains physical functioning and physical component summary. These findings are in concordance with the findings of others, as age and the duration of symptoms of spinal cord disease have been associated with a higher degree of disability [5, 9, 7].

Moreover, aside from being the largest follow-up study in women with ALD to date, this is also the first study to use a semi-targeted lipidomics approach in plasma for the identification of new diagnostic biomarkers for ALD in women. Our approach generated a list of 100 potential biomarker ratios with strong differentiating properties and non-overlapping data distributions between women with ALD (*n* = 20) and controls (*n* = 10). As the selected group of women with ALD included five women with either a plasma C26:0 level or a C26:0/C22:0 ratio within the normal range, these results make us feel confident that our list of potential biomarker ratios will most likely contain a ratio with a better sensitivity than plasma C26:0 levels or the C26:0/C22:0. Using a comparable lipidomics approach Ruiz et al. (2015) reported similar differences in abundancy of lipids between 13 men with ALD and 13 controls [33]. The finding that the majority of the 56 potential new biomarkers belong to the phosphatidylcholine class was not unexpected. Already in the 90’s it was reported that in normal-appearing white matter the highest VLCFA excess was found in the phosphatidylcholine fraction [34]. After validation of our results in an external or independent cohort we will make a selection of ratios for further exploration based on feasibility, the availability of internal standards and absolute abundance of the individual metabolites. Thereafter a dedicated method can be put into place for use in clinical care.

## Conclusions

To summarize, progression after 8-year follow-up was detectable in our cohort using the EDSS, but the change in EDSS score was small. These results have implications for counseling women with ALD. These women may be informed that progression is usually very slow, with significant change occurring over years or decades, although in individual patients onset may be early and the disease more progressive. Why some women become symptomatic decades before other women or which factors influence the rate of progression remains to be elucidated in future studies. Furthermore, including women in intervention trials with clinical endpoints evaluating new treatments for spinal cord disease in ALD remains challenging. Progression seems so slow that it cannot be detected by current outcome measures such as EDSS, ALDS and SF-36 unless a study lasts for at least 8 years, which is typically not feasible from a financial perspective. Perhaps clinical outcome measures primarily focused on gait (e.g. six-minute walk test or the spastic paraplegia rating scale), sensory abnormalities (e.g. semi-quantitative vibration threshold) or incontinence (e.g. International Consultation on Incontinence Questionnaire Female Lower Urinary Tract Symptoms) can detect a more significant change between baseline and follow-up, but additional, more sensitive, quantitative measures for progression of spinal cord disease are needed to detect disease progression during a smaller and thus more practical time period [19, 35–37]. Current candidates are optical coherence tomography and spinal cord DTI [38–40], however, these modalities remain to be validated in future studies.

## Additional files

Additional file 1: Individual clinical data: mutations, symptoms and signs. The presence of symptoms or signs is categorized in 1 ‘Yes’ and 0 ‘No’. The duration of neurological symptoms is categorized in 0 ‘ No myelopathy ’, 1 ‘ Neurological symptoms for ≤ 10 years ’ and 2 ‘ Neurological symptoms for > 10 years ’. (XLS 50 kb)

Additional file 2: Individual clinical data: SF-36, EDSS and ALDS values. SF-36 values were compared with norm values for the Dutch population and corrected for gender and age. The SF-36 subdomain scores are expressed in Z scores, ranging from − 4 (lowest quality of life) to + 4 (highest quality of life). The SF-36 summary scores were linearly transformed to a range from 0 (lowest quality of life) to 100 (highest quality of life), with a mean of 50 and standard deviation of 10. The EDSS ranges from 0 (normal) to 10 (death). The ALDS scores are regression coefficients which were linearly transformed for interpretation, ranging from 10 (highest level of disability) to 89.47 (lowest level of disability). ALDS = AMC Linear Disability Scale; EDSS = Expanded Disability Status Scale; MCS = mental component summary; PCS = physical component summary; SF-36 = Short Form (36) Health Survey. (XLSX 21 kb)

Additional file 3: Construct validity. Spearman’s rank-order correlation were run to assess correlations between the outcome measures. A Bonferroni correction was applied for multiple comparisons. Correlations were considered significant at the level of 0.0042 (2-tailed). * indicates significant correlations. ALDS = AMC Linear Disability Scale; BP = bodily pain; EDSS = Expanded Disability Status Scale; GH = general health perceptions; MCS = mental component summary; MH = mental health; PCS = physical component summary; PF = physical functioning; r = correlation coefficient; RE = role limitations due to emotional problems; RP = role limitations due to physical problems; SF = social functioning; VT = vitality. (DOC 70 kb)

## Abbreviations

ALD: X-linked adrenoleukodystrophy
ALDP: ALD protein
AMN: Adrenomyeloneuropathy
B: Coefficient
BMP: Bis(monoacylglycero)phosphate
BP: Bodily pain
C26:0-lysoPC: 1-hexacosanoyl-2-lyso-sn-3-glycero-phosphorylcholine
CE: Cholesterol ester
Cer: Ceramide
CL: Cardiolipin
DG: Diglycerides
EDSS: Expanded Disability Status Scale
GH: General health perceptions
H: Kruskal-Wallis H statistic
JOA: Japanese Orthopaedic Association
LPA: Lysophosphatidic acid
LPC: Lysophosphatidylchloline
LPE: Lysophosphatidylethanolamine
LPG: Lysophosphatidylglycerol
MCS: Mental component summary
MH: Mental health
N: Number of patients
PA: Phosphatidic acid
PC: Phosphatidylcholine
PCS: Physical component summary
PE: Phosphatidylethanolamine
PF: Physical functioning
PG: Phosphatidylglycerol
PI: Phosphatidylinositol
PLS-DA: Partial least squares regression discriminant analysis
PS: Phosphatidylserine
r: Correlation coefficient
RE: Role limitations due to emotional problems
Role emotional: Role limitations due to emotional problems
Role physical: Role limitations due to physical problems
RP: Role limitations due to physical problems
SE: Standard error
SF: Social functioning
SF-36: Short Form (36) Health Survey
SM: Sphingomyelin
SSPROM: Severity Score system for Progressive Myelopathy
TG: Triglycerides
U: Mann-Whitney U statistic
UPLC-HRMS: Ultra-high performance liquid chromatography coupled to high-resolution mass spectrometry
VIP: Variable importance of projection
VLCFA: Very long-chain fatty acids
VT: Vitality
