# Supplementary material for: Disease progression in women with X-linked adrenoleukodystrophy is slow
Source: Orphanet J Rare Dis. 2019 Feb 7;14:30. doi: 10.1186/s13023-019-1008-6 (PMC6367840; doi:10.1186/s13023-019-1008-6)
Supplement: Supplementary file 3 — Construct validity. Spearman’s rank-order correlation were run to assess correlations between the outcome measures. A Bonferroni correction was applied for multiple comparisons. Correlations were considered significant at the level of 0.0042 (2-tailed). * indicates significant correlations. ALDS = AMC Linear Disability Scale; BP = bodily pain; EDSS = Expanded Disability Status Scale; GH = general health perceptions; MCS = mental component summary; MH = mental health; PCS = physical component summary; PF = physical functioning; r = correlation coefficient; RE = role limitations due to emotional problems; RP = role limitations due to physical problems; SF = social functioning; VT = vitality. (DOC 70 kb) [file 13023_2019_1008_MOESM3_ESM.doc]

|  | | **EDSS** | **ALDS** | **PF** | **RP** | **BP** | **GH** | **VT** | **SF** | **RE** | **MH** | **PCS** | **MCS** |
| --- | --- | --- | --- | --- | --- | --- | --- | --- | --- | --- | --- | --- | --- |
| **EDSS** | **r** |  | -.70* | -.50* | -0.23 | -.27 | -.29 | -0.10 | -0.14 | 0.08 | -0.03 | -.41* | 0.11 |
|  | **p** |  | 0.00 | 0.00 | 0.07 | 0.03 | 0.02 | 0.44 | 0.27 | 0.53 | 0.85 | 0.00 | 0.41 |
| **ALDS** | **r** | -.70* |  | .65* | .54* | .56* | .50* | .28* | .35* | 0.13 | 0.01 | .64* | -0.06 |
|  | **p** | 0.00 |  | 0.00 | 0.00 | 0.00 | 0.00 | 0.03 | 0.00 | 0.32 | 0.98 | 0.00 | 0.66 |
| **PF** | **r** | -.50* | .65* |  | .66* | .67* | .77* | .51* | .56* | 0.20 | 0.20 | .89* | -0.12 |
|  | **p** | 0.00 | 0.00 |  | 0.00 | 0.00 | 0.00 | 0.00 | 0.00 | 0.11 | 0.13 | 0.00 | 0.37 |
| **RP** | **r** | -0.23 | .54* | .66* |  | .70* | .58* | .43* | .62* | .42* | 0.22 | .74* | 0.08 |
|  | **p** | 0.07 | 0.00 | 0.00 |  | 0.00 | 0.00 | 0.00 | 0.00 | 0.00 | 0.08 | 0.00 | 0.54 |
| **BP** | **r** | -.27 | .56* | .67* | .70* |  | .69* | .51* | .54* | 0.21 | 0.14 | .87* | -0.17 |
|  | **p** | 0.03 | 0.00 | 0.00 | 0.00 |  | 0.00 | 0.00 | 0.00 | 0.10 | 0.26 | 0.00 | 0.18 |
| **GH** | **r** | -.29 | .50* | .77* | .58* | .69* |  | .57* | .66* | .26 | .32 | .85* | 0.05 |
|  | **p** | 0.02 | 0.00 | 0.00 | 0.00 | 0.00 |  | 0.00 | 0.00 | 0.04 | 0.01 | 0.00 | 0.68 |
| **VT** | **r** | -0.10 | .28 | .51* | .43* | .51* | .57* |  | .54* | .28 | .39* | .51* | .39* |
|  | **p** | 0.44 | 0.03 | 0.00 | 0.00 | 0.00 | 0.00 |  | 0.00 | 0.03 | 0.00 | 0.00 | 0.00 |
| **SF** | **r** | -0.14 | .35* | .56* | .62* | .54* | .66* | .54* |  | .42* | .38* | .59* | .37* |
|  | **p** | 0.27 | 0.00 | 0.00 | 0.00 | 0.00 | 0.00 | 0.00 |  | 0.00 | 0.00 | 0.00 | 0.00 |
| **RE** | **r** | 0.08 | 0.13 | 0.20 | .42* | 0.21 | .26 | .28 | .42* |  | .40* | 0.14 | .56* |
|  | **p** | 0.53 | 0.32 | 0.11 | 0.00 | 0.10 | 0.04 | 0.03 | 0.00 |  | 0.00 | 0.27 | 0.00 |
| **MH** | **r** | -0.03 | 0.01 | 0.20 | 0.22 | 0.14 | .32 | .39* | .38* | .40* |  | 0.08 | .70* |
|  | **p** | 0.85 | 0.93 | 0.13 | 0.08 | 0.26 | 0.01 | 0.00 | 0.00 | 0.00 |  | 0.55 | 0.00 |
| **PCS** | **r** | -.41* | .64* | .89* | .74* | .87* | .85* | .51* | .59* | 0.14 | 0.08 |  | -0.24 |
|  | **p** | 0.00 | 0.00 | 0.00 | 0.00 | 0.00 | 0.00 | 0.00 | 0.00 | 0.27 | 0.55 |  | 0.06 |
| **MCS** | **r** | 0.11 | -0.06 | -0.12 | 0.08 | -0.17 | 0.05 | .39* | .37* | .56* | .70* | -0.24 |  |
|  | **p** | 0.41 | 0.66 | 0.37 | 0.54 | 0.18 | 0.68 | 0.00 | 0.00 | 0.00 | 0.00 | 0.06 |  |

Additional file 3. Construct validity

**Spearman's rank-order correlation were run to assess correlations between the outcome measures. A Bonferroni correction was applied for multiple comparisons. Correlations were considered significant at the level of 0.0042 (2-tailed). * indicates significant correlations. ALDS = AMC Linear Disability Scale; BP = bodily pain; EDSS = Expanded Disability Status Scale; GH = general health perceptions; MCS = mental component summary; MH = mental health; PCS = physical component summary; PF = physical functioning; r = correlation coefficient; RE = role limitations due to emotional problems; RP = role limitations due to physical problems; SF = social functioning; VT = vitality.**
